# Multi-Omics Analysis Reveals the Potential Role of *Inonotus obliquus* in Mit-Igating Hemolytic Jaundice

**DOI:** 10.3390/nu18193225

**Published:** 2026-09-29

**Authors:** Fan Yang, Siyi Xie, Wenjing Yang, Chuanhong Zhu, Yurong Chen, Haozhuo Yang, Hongxia Yuan, Qingshan Li

**Affiliations:** 1 Medicinal Basic Research Innovation Center of Chronic Kidney Disease, Ministry of Education, School of Pharmacy, Shanxi Medical University, Jinzhong 030600, China; fanfan42@sxmu.edu.cn (F.Y.); xsy1213@b.sxmu.edu.cn (S.X.); yvette0919@b.sxmu.edu.cn (W.Y.); 202500611114@sxmu.edu.cn (C.Z.); 202500631116@sxmu.edu.cn (Y.C.); 202500631168@sxmu.edu.cn (H.Y.); 2 Shanxi Key Laboratory of Innovative Drug for the Treatment of Serious Diseases Basing on the Chronic Inflammation, College of Traditional Chinese Medicine and Food Engineering, Shanxi University of Chinese Medicine, Jinzhong 030619, China

**Keywords:** *Inonotus obliquus*, hemolytic jaundice, gut microbiota, gut-liver axis

## Abstract

**Background:** Hemolytic jaundice, characterized by excessive bilirubin production and hepatic dysfunction, currently lacks effective therapeutic strategies targeting its underlying mechanisms. **Methods:** This study evaluated the therapeutic potential of the aqueous extract of *Inonotus obliquus* (IO) in ameliorating hemolytic jaundice through modulation of the gut-liver axis. A murine model of hemolytic jaundice was induced by phenylhydrazine (PHZ) administration, and IO was orally administered as the intervention. Antibiotic depletion and FMT verified gut microbiota dependence. Liver injury, serum bilirubin, gut microbiome, and metabolites were assessed. **Results:** IO treatment significantly alleviated PHZ-induced hemolytic jaundice in mice. Notably, antibiotic-mediated depletion of the gut microbiota abolished the hepatoprotective effects of IO, whereas fecal microbiota transplantation (FMT) from IO-treated donors conferred marked amelioration of hyperbilirubinemia in recipient mice. Integrative multi-omics analysis identified Limosilactobacillus reuteri and Lactobacillus johnsonii as the predominant microbial species altered by IO. Furthermore, IO upregulated the expression of farnesoid X receptor (FXR) and pregnane X receptor (PXR) in both hepatic and intestinal tissues and also increased the expression of bile salt export pump (BSEP) and multidrug resistance-associated protein 2 (MRP2). These alterations were correlated with an increased excretion of bile acids and bilirubin into the intestinal lumen, thereby mitigating hepatic injury. This process may be associated with the modulation of gut microbiota. **Conclusions**: These findings collectively provide evidence of a significant correlation between IO treatment and the mitigation of hemolytic jaundice, achieved by modulating the enterohepatic circulation of bile acids and bilirubin through the regulation of gut microbiota. Our results highlight the potential of IO as a microbiota-directed nutritional strategy for the management of jaundice.

## 1. Introduction

Neonatal jaundice, or neonatal hyperbilirubinemia, is a common condition in newborns, characterized by yellowing of the skin, mucous membranes, and eyes [1]. It results from an imbalance in bilirubin metabolism, including its production, processing, and excretion. Causes include infections, hemolytic disease of the newborn (HDN), and congenital biliary issues [2]. HDN is the leading cause, as it involves rapid red blood cell breakdown, releasing too much bilirubin for the liver to handle, causing jaundice [3]. The most severe potential complication of this condition is bilirubin neurotoxicity (kernicterus), which can cause irreversible neurological damage, including cerebral palsy and sensorineural hearing loss [4]. Current primary clinical interventions are phototherapy (blue light exposure) and exchange transfusion [5]. However, these approaches have significant limitations: high cost, notable side effects, and variable efficacy, with particularly poor outcomes in infants who respond inadequately to phototherapy [6]. Notably, even after conventional treatment, approximately 30% of infants with hemolytic jaundice exhibit persistent liver function abnormalities [7]. This observation underscores a key deficiency in current therapeutic strategies: their inadequacy in effectively regulating the bilirubin enterohepatic cycle.

The liver functions as the principal site for bilirubin metabolism [8], where unconjugated bilirubin (UCB) undergoes conjugation to form conjugated bilirubin (CB), which is then excreted into the intestinal tract via bile [9]. Within the gut, a fraction of CB is hydrolyzed back into UCB by microbial-derived β-glucuronidase (β-GD), facilitating its reabsorption into the bloodstream and consequently augmenting the bilirubin load [10]. The remaining fraction is further reduced by the gut microbiota into urobilinogen and stercobilin, which are ultimately excreted in urine and feces [11]. Neonates are particularly vulnerable to bilirubin accumulation due to the low hepatic activity of UDP-glucuronosyltransferase (UGT1A1) and an active enterohepatic circulation [12].

Recent advancements in microbiome research have increasingly clarified the role of gut microbiota in bilirubin metabolism through the gut-liver axis [13], providing novel insights for microbial interventions in the treatment of jaundice. Empirical studies suggest that children with hemolytic jaundice demonstrate significant alterations in the composition of their gut microbiota. These alterations are characterized by an overgrowth of opportunistic pathogens, such as Enterobacteriaceae, and a notable decrease in protective Bifidobacterium populations. Certain strains of Bifidobacterium, such as B. bifidum, have been shown to modulate bilirubin metabolism through a complex “gut microbiota–linoleic acid metabolism–enterohepatic circulation” axis, thereby contributing to improved liver function and enhanced intestinal barrier integrity [14]. Additionally, studies on cholestatic liver disease indicate that gut microbiota may also affect bile acid metabolism by regulating bile salt transporters such as Na^+^-taurocholate cotransporting polypeptide (Ntcp) and Bile Salt Export Pump (BSEP), thereby mitigating cholestasis [15]. Overall, these results underscore the significance of microbiota-host interactions in the therapeutic management of jaundice.

*Inonotus obliquus* (IO), commonly known as the Chaga mushroom, is a medicinal fungus that predominantly parasitizes birch trees and is primarily distributed across cold regions of the Northern Hemisphere, including Siberia, Northern Europe, North America, and Northeast China [16]. The use of IO in traditional medicine dates to the 16th and 17th centuries in Eastern Europe and Russia, where it has been employed for the treatment of digestive disorders, diabetes, cardiovascular diseases, and cancer [17]. The therapeutic potential of IO is attributed to its rich composition of bioactive constituents, notably polysaccharides, betulin, triterpenoids, and polyphenols [18]. While its antitumor, antidiabetic, antioxidant, and anti-inflammatory properties are well-established [19], recent scientific interest has pivoted towards its potential to modulate the gut microbiota, a crucial mediator of human health [20]. This prebiotic-like activity is a shared characteristic among many medicinal fungi. The polysaccharides present in IO are of academic interest, as they are considered among its most significant bioactive constituents. Emerging research underscores their role in modulating gut microbiota, a critical aspect of the gut ecosystem [21,22]. This modulation is essential for harnessing the full potential of IO in functional food applications designed to improve gut health and human wellness.

Although intricate connections exist among IO, gut microbiota, and bilirubin metabolism, the therapeutic potential of IO in hemolytic jaundice and its modulatory influence on the gut microbiome have not been investigated. Therefore, in this study, utilizing a well-established PHZ-induced murine model of hemolytic jaundice, we evaluated the therapeutic effects of oral IO administration. Our findings demonstrated a significant attenuation of jaundice and liver injury by IO. Crucially, this protective effect was entirely abolished upon depletion of the gut microbiota using broad-spectrum antibiotics, establishing a fundamental dependence on gut microbes. Furthermore, fecal microbiota transplantation (FMT) from IO-treated mice conferred protection against PHZ-induced jaundice in recipient mice, directly implicating the gut microbiota in mediating IO’s benefits. Our results are designed to offer scientific support for the use of IO as a supportive therapeutic approach in hemolytic jaundice.

## 2. Materials and Methods

### 2.1. Chemicals and Reagents

Phenylhydrazine hydrochloride (PHZ, Cat# 114715) was purchased from sigma-Aldrich (St. Louis, MO, USA). Assay kits for alanine aminotransferase (ALT), aspartate aminotransferase (AST), total bilirubin (TBIL) and direct bilirubin (DBIL) were all purchased from Nanjing Jiancheng Bioengineering Institute (Nanjing, China). Yinzhihuang Oral Liquid was purchased from Beijing Huarun High-Tech Natural Medicine Co., Ltd. (Beijing, China), with batch number 240808. Antibody for β-actin (Cat# AC016, 1:10,000; RRID: AB_2863784, alternative clone AC038) was purchased from Abclonal (Woburn, MA, USA). Antibody for farnesoid X receptor (FXR) (Cat# 25055-1-AP, 1:2000; RRID: AB_2879874) was purchased from Proteintech (Rosemont, IL, USA). Antibodies for pregnane X receptor (PXR) (Cat# DF6478, 1:1000; RRID: Not assigned by vendor), BSEP (Cat# AF7593, 1:1000; RRID: Not assigned by vendor), multidrug resistance-associated protein 2 (MRP2) (Cat# DF3873, 1:1000; RRID: AB_2836226) were purchased from Affinity Biosciences (Cincinnati, OH, USA). Metronidazole (M109874), ampicillin (A102048), vancomycin (V301569), and neomycin sulfate (N412785) were purchased from Aladdin Industrial Corporation (Shanghai, China). Phenol and carbazole were obtained from Tianjin Fengchuan Chemical Reagent Technology Co., Ltd. (Tianjin, China). Concentrated sulfuric acid was supplied by Beijing Chemical Works (Beijing, China). D-glucose was purchased from Beijing Hengyuan Qitian Chemical Technology Research Institute (Beijing, China). D-galacturonic acid was provided by the National Institutes for Food and Drug Control (NIFDC, Beijing, China). Sodium tetraborate was obtained from Sinopharm Chemical Reagent Co., Ltd. (Shanghai, China). 3,5-Dinitrosalicylic acid (DNS) reagent was acquired from Fuzhou Webekang Biotechnology Co., Ltd. (Fuzhou, China). Folin–Ciocalteu reagent and gallic acid were purchased from Macklin Biochemical Co., Ltd. (Shanghai, China). Sodium carbonate was supplied by Tianjin Huasheng Chemical Reagent Co., Ltd. (Tianjin, China). The BCA protein quantification kit was obtained from Solarbio Science & Technology Co., Ltd. (Beijing, China). All reagents were of analytical grade unless otherwise specified.

### 2.2. Extract Preparation/Storage/Stability

IO raw material was procured from Daxing’anling Senyuan Mountain Products Co., Ltd. (Daxing’anling, China). The fungal raw material was crushed into coarse granules, soaked at a solid–liquid ratio of 1:10 (*w*/*v*) for 3 h, and subsequently boiled at 100 °C for 2 h. After centrifugation, the residual pellet was re-extracted twice under identical conditions for 1.5 h each time. All supernatants obtained from the three extraction cycles were combined. Anhydrous ethanol was then added to the pooled supernatants to achieve a final ethanol concentration of 80% (*v*/*v*). The mixture was kept stationary at 4 °C for 12 h for ethanol precipitation, and precipitates were harvested by centrifugation. The precipitates were vacuum-dried at 0.07 MPa and 55 ± 1 °C to obtain IO extract powder [23]. Briefly, 100.5 g of raw IO material was weighed for extraction, and 13.18 g of dried extract powder was ultimately obtained, corresponding to an extraction yield of 13.10% (*w*/*w*). All IO extract batches were stored at −20 °C in amber vials and working solutions for animal administration were freshly prepared on each day of use to minimize degradation. Three independent batches of IO extract were prepared under identical extraction conditions following our previously established protocols. The extraction yield and the levels of major components (polysaccharides, uronic acid, total protein, and total phenols) showed low inter-batch standard deviations, confirming good reproducibility and stable chemical composition.

### 2.3. Qualitative Analysis of IO

#### 2.3.1. Total Polysaccharides

Total Polysaccharides were quantified via the phenol–sulfuric acid method using D-glucose as the reference standard [24]. Briefly, gradient concentrations of D-glucose standard solution were prepared. Equal volumes of sample/standard solution, 5% phenol solution and concentrated sulfuric acid were mixed thoroughly. The mixture was incubated in an 80 °C water bath for 30 min. After cooling to ambient temperature, absorbance was measured at 486 nm to establish the total sugar standard curve. For sample testing, the extract was dissolved in deionized water to prepare 0.2 mg/mL sample solution and measured following identical procedures.

#### 2.3.2. Total Reducing Sugar

Reducing sugar was detected using the DNS colorimetric method [25]. Serial dilutions of D-glucose standard solution were mixed with DNS reagent, heated in a boiling water bath for 5 min, cooled rapidly and diluted to a constant volume. Absorbance was recorded at 540 nm to plot the reducing sugar calibration curve. For sample testing, the extract was dissolved in deionized water to prepare 0.2 mg/mL sample solution and measured following identical procedures.

#### 2.3.3. Uronic Acids

Uronic acid content was determined by the carbazole–sulfuric acid assay [26]. Briefly, the extract powder was dissolved in ultrapure water. Sodium tetraborate–sulfuric acid solution was added under an ice-water bath, followed by incubation in a boiling water bath for 20 min. After adding 0.1% carbazole ethanol solution, the mixture was kept at room temperature for 2 h. The absorbance was detected at 530 nm, and galacturonic acid was used to generate the standard curve for quantitative calculation.

#### 2.3.4. Total Protein

Protein content was measured using the BCA method. Briefly, sample solution (25 μL) was transferred into a 96-well plate, mixed with 200 μL BCA working solution per well, and incubated at 37 °C for 30 min. The absorbance at 562 nm was determined, and protein concentration was calculated based on the standard curve supplied with the BCA kit.

#### 2.3.5. Total Phenols

Total phenolic content was analyzed using an optimized Folin–Ciocalteu method [27]. In brief, sample solution was mixed with Folin–Ciocalteu reagent and incubated for 5 min. Subsequently, 7.5% sodium carbonate solution was added, and the mixture was kept in the dark at room temperature for 60 min. Absorbance was measured at 765 nm, and gallic acid was applied as the standard to calculate total phenolic content.

#### 2.3.6. UPLC-MS/MS Method

For qualitative analysis, 30 mg of IO powder was accurately weighed and mixed with 1500 μL of a −20 °C 70% methanol solution with internal standards, followed by vortexing and centrifugation. The final extract was obtained by filtering the supernatant. UPLC-MS/MS analysis was carried out using an Agilent SB-C18 column (Agilent Technologies, Santa Clara, CA, USA) and a water-acetonitrile gradient.

### 2.4. Animal

Male ICR mice (6–8 weeks old) were provided by Sibeifu (Beijing) Biotechnology Co., Ltd. (Beijing, China) (Certificate No. SCXK (Jing) 2024-0001). Endogenous estrogen in female mice suppresses the FXR/PXR signaling cascade and remodels bile acid profiles as well as gut microbiota composition, which would confound the mechanistic outcomes of this research [28]. In the PHZ-induced hemolysis model, female mice exhibit highly variable biochemical indices and poor experimental reproducibility [29,30]. Furthermore, estrous cycle synchronization cannot be achieved across all groups within our complicated experimental design, which would substantially increase experimental bias [31]. Consistent with previous studies on *Inonotus obliquus* extracts, gut-liver axis metabolism, and PHZ-triggered hemolytic injury, male rodents were selected to eliminate confounding effects derived from sex hormones and ensure stable, comparable experimental data [32,33]. The animals were housed under standard conditions at the Animal Experiment Center of Shanxi Medical University. All experimental procedures were approved by the Institutional Animal Care and Use Committee and conducted in accordance with the relevant guidelines. All experimental protocols were reviewed and approved by the Medical Ethics Committee of Shanxi University of Traditional Chinese Medicine, Taiyuan, China (AWE202209151).

Sample size was determined by an a priori power analysis using G*Power software (version 3.1.9.6) for an independent two-group *t*-test. Since no suitable effect size was available from published literature for this specific model, we estimated the effect size from our pilot experiment based on serum total bilirubin levels (*n* = 5 per group; control: 3.92 ± 2.66, PHZ: 24.63 ± 10.59, Cohen’s d = 2.68). We adopted a conservative effect size of d = 2.0 to ensure a more stringent sample size calculation. With a two-sided significance level of α = 0.05 and a statistical power of 95% (1 − β = 0.95), the minimum sample size required was 8 mice per group. To account for potential dropouts and experimental variability, 10 mice per group were used in the formal experiment. The number of animals was minimized in accordance with the 3R principle, and all experimental protocols were reviewed and approved by the Medical Ethics Committee of Shanxi University of Traditional Chinese Medicine (approval code AWE202209151).

### 2.5. Animal Modeling and Processing

#### 2.5.1. Hemolytic Jaundice Model and IO Treatment

Mice were randomly assigned to six groups (*n* = 10 per group): the control group (CON, Ctrl), the model group (MOD, PHZ), the PHZ+IO-L group (IO-L, 200 mg/kg/d), the FHZ+IO-M group (IO-M, 300 mg/kg/d), the PHZ+IO-H group (IO-H, 400 mg/kg/d), and the PHZ+Yingzhihuang group (YZH, 10 mL/kg/d). The hemolytic jaundice mouse model was created by administering two intraperitoneal injections of 75 mg/kg PHZ solution [34]. The model control group and the control group received 10 mL/kg/d of saline by oral gavage, once daily for 5 consecutive days.

#### 2.5.2. Antibiotic Depletion Experiment

An intestinal sterile mouse model was established in twenty mice by administering an antibiotic cocktail (ampicillin, 1 mg/mL; neomycin, 1 mg/mL; metronidazole, 1 mg/mL; vancomycin, 0.5 mg/mL) in drinking water, which was replaced daily [35]. After 14 days, the model was induced with PHZ. Mice were then randomized into two groups: the ABX-PHZ group received normal saline, and the ABX-IO group received IO (400 mg/kg/d) for 5 days. All mice were euthanized on the 6th day by gradual introduction of CO_2_ (20–30% chamber volume/min) followed by cervical dislocation to ensure death. The euthanasia procedure was performed in accordance with the guidelines approved by the Institutional Animal Care and Use Committee, and all efforts were made to minimize animal suffering.

#### 2.5.3. Fecal Microbiota Transplantation Experiment

Fecal microbiota transplantation was performed as follows. Following 14 days of antibiotic preconditioning in drinking water to establish a pseudo-germ-free mouse model as described in Section 2.5.2. The mice were randomly assigned to two groups: the FMT-PHZ group and the FMT-IO group, which received fecal transplants from the model group and the treatment group, respectively. Donor feces were collected from PHZ model mice and PHZ+IO (400 mg/kg/d) mice, established as per Section 2.5.1. The feces were homogenized in sterile saline to a concentration of 0.2 g/mL. The supernatant was collected after centrifugation and filtration and then administered to mice in the FMT-PHZ and FMT-IO groups (100 µL/d via oral gavage for 10 days) [36]. After 10 days, mice in both groups were intraperitoneally injected with PHZ to induce the model, while FMT was continued for another 5 days. Mice in the CON group were given antibiotic water for 14 days, then switched to normal water and gavaged daily with saline (10 mL/kg/d) until euthanasia. All mice were euthanized on the 18th day.

### 2.6. Intestinal and Hepatic Pathology

Following collection, liver and colon tissues of suitable size were fixed in 4% paraformaldehyde, dehydrated, and embedded in paraffin. Sections were then prepared, stained, dehydrated, and mounted for light microscopic examination of hepatocyte morphology. All histological evaluations were performed in a blinded manner, with the assessors unaware of the treatment group assignments. Quantification of histological features was conducted using ImageJ software (version 2.14.0/1.54f) under identical threshold settings for all images to ensure objectivity and reproducibility.

### 2.7. Detection of Hepatocyte Apoptosis by TUNEL Assay

Following pretreatment steps including deparaffinization, proteinase digestion, and PBS rinsing, liver paraffin sections were incubated according to the instructions of the TUNEL staining kit. After staining, the sections were observed under a fluorescence microscope, and the percentage of fluorescence-positive cells was quantified using ImageJ software.

### 2.8. 16S rRNA Sequencing Analysis

Metabolites from snap-frozen fecal samples (80 mg) were extracted via homogenization in water followed by liquid–liquid extraction with cold methanol/acetonitrile (1:1, *v*/*v*). Following centrifugation and vacuum drying, the extract was reconstituted in acetonitrile/water (1:1, *v*/*v*) for analysis. Separation utilized a HILIC column with an acetonitrile/aqueous ammonium solvent gradient. Detection was performed on two platforms: a Q-TOF system (AB Sciex, Framingham, MA, USA; *m*/*z* 60–1000; ISVF ±5500 V; 600 °C) and an Orbitrap system (Thermo Fisher Scientific, Waltham, MA, USA; *m*/*z* 70–1200; Resolution 60,000; ISVF +3500/−2800 V; 350 °C).

### 2.9. Metabolome Analysis

Metabolites were extracted from 80 mg of snap-frozen feces by homogenization in water followed by methanol/acetonitrile (1:1) extraction, centrifugation, and vacuum drying. The extract was reconstituted in acetonitrile/water (1:1) for LC-MS analysis. Separation was performed on a HILIC column with an acetonitrile/aqueous ammonium gradient. Mass spectrometry was conducted on two systems: a Q-TOF instrument (AB Sciex; *m*/*z* 60–1000; ISVF ±5500 V; 600 °C) and an Orbitrap instrument (Thermo Fisher Scientific, Waltham, MA, USA; *m*/*z* 70–1200; Resolution 60,000; ISVF +3500/−2800 V; 350 °C).

### 2.10. Short-Chain Fatty Acid Analysis

Short-chain fatty acids (SCFAs) were analyzed from fecal samples. Briefly, 20 mg of feces was homogenized in 0.5% phosphoric acid, and the supernatant was liquid–liquid extracted with MTBE (containing IS) after centrifugation. Analysis was performed on an Agilent 7890B-7000D GC-MS/MS system (Agilent Technologies, Santa Clara, CA, USA) with a DB-FFAP column using a programmed temperature gradient and helium carrier gas (1.2 mL/min). Injection details: split mode (5:1), 1 μL volume, 230 °C inlet temperature. All chemicals and standards were sourced from CNW or Aladdin.

### 2.11. Metagenomic Analysis

Metagenomic sequencing was performed as follows. DNA samples meeting the quality control thresholds (concentration > 0.2 μg as measured by Qubit 4.0, and fragment size > 500 bp as assessed by Qsep400 Bioanalyzer) were used for library construction. Using 0.2 μg of DNA as input, samples were fragmented to an average size of 300 bp, followed by end repair, A-tailing, and adapter ligation for Illumina sequencing. The resulting libraries were amplified via PCR, purified, and their size distribution was confirmed. Finally, the prepared libraries were pooled, denatured, and loaded onto a flow cell for sequencing on the Illumina platform.

### 2.12. RT-qPCR

Total RNA was isolated from mouse liver and colon tissues using the Trizol reagent. RNA was reverse transcribed into cDNA using the PrimeScript™ RT reagent kit with gDNA Eraser (Takara, Beijing, China). qPCR was performed on a LightCycler 480 II instrument (Roche, Indianapolis, IN, USA) using SYBR Green Supermix (EZB, Roseville, CA, USA). The relative expression levels of target mRNAs were calculated via the ΔΔCt method, with *GAPDH* serving as the reference gene for normalization of Ct values. The primer sequences utilized are provided in Supplementary Table S1.

### 2.13. Western Blot

An appropriate amount of RIPA lysis buffer was added to colon and liver tissues, which were then thoroughly homogenized using an SWE-FP tissue grinder (Servicebio, Wuhan, China). After determining the protein concentration of the supernatant via the BCA assay, 20 μg of protein was loaded for SDS-PAGE electrophoresis. Following electrophoresis, proteins were transferred onto a PVDF membrane, which was subsequently blocked with 5% skim milk for 2 h. The membrane was incubated overnight at 4 °C with primary antibodies targeting the proteins of interest, followed by incubation with secondary antibodies at room temperature for 1 h. After washing three times with TBST buffer, ECL luminescence solution was applied. Protein bands were visualized using the GelDoc Go gel imaging system (Bio-Rad Laboratories, Inc. Hercules, CA, USA), and gray-level analysis was performed with ImageJ software.

### 2.14. Statistical Analysis

Data were analyzed using GraphPad Prism 9.5 software (GraphPad Software, Inc., San Diego, CA, USA). An unpaired Student’s *t*-test was conducted for comparisons between two groups, while one-way analysis of variance (ANOVA) was used for comparisons among multiple groups. Results are presented as mean ± standard deviation (Mean ± SD), with *p* < 0.05 considered statistically significant.

Sample size was determined by an a priori power analysis using G*Power software (version 3.1.9.6) using the “Means: Difference between two independent means (two groups)” option for a two-tailed independent *t*-test. Based on pilot data (*n* = 5 per group; control: 3.92 ± 2.66, PHZ: 24.63 ± 10.59, Cohen’s d = 2.68), a conservative effect size of d = 2.0 was adopted. With a two-sided α = 0.05 and power = 0.95, the minimum sample size required was 8 mice per group. To account for potential dropouts, 10 mice per group were used in the formal experiment.

For microbiome analysis, alpha diversity (Shannon and Chao1) was compared using Kruskal–Wallis with Dunn’s post hoc test, and beta diversity was assessed by PCoA based on Bray–Curtis distances with PERMANOVA (999 permutations). Differential genera were identified using LEfSe (LDA > 2, *p* < 0.05) and DESeq2 (FDR-adjusted *p* < 0.05). For metabolomic data, OPLS-DA was performed using MetaboAnalyst 5.0, with differential metabolites selected by VIP > 1 and *p* < 0.05 (*t*-test). Pathway enrichment was analyzed via KEGG using hypergeometric test, with *p* < 0.05 considered significant. Multiple comparisons were corrected using FDR where applicable.

## 3. Results

### 3.1. Content Determination and Component Identification in IO Extracts

**Figure 1:**
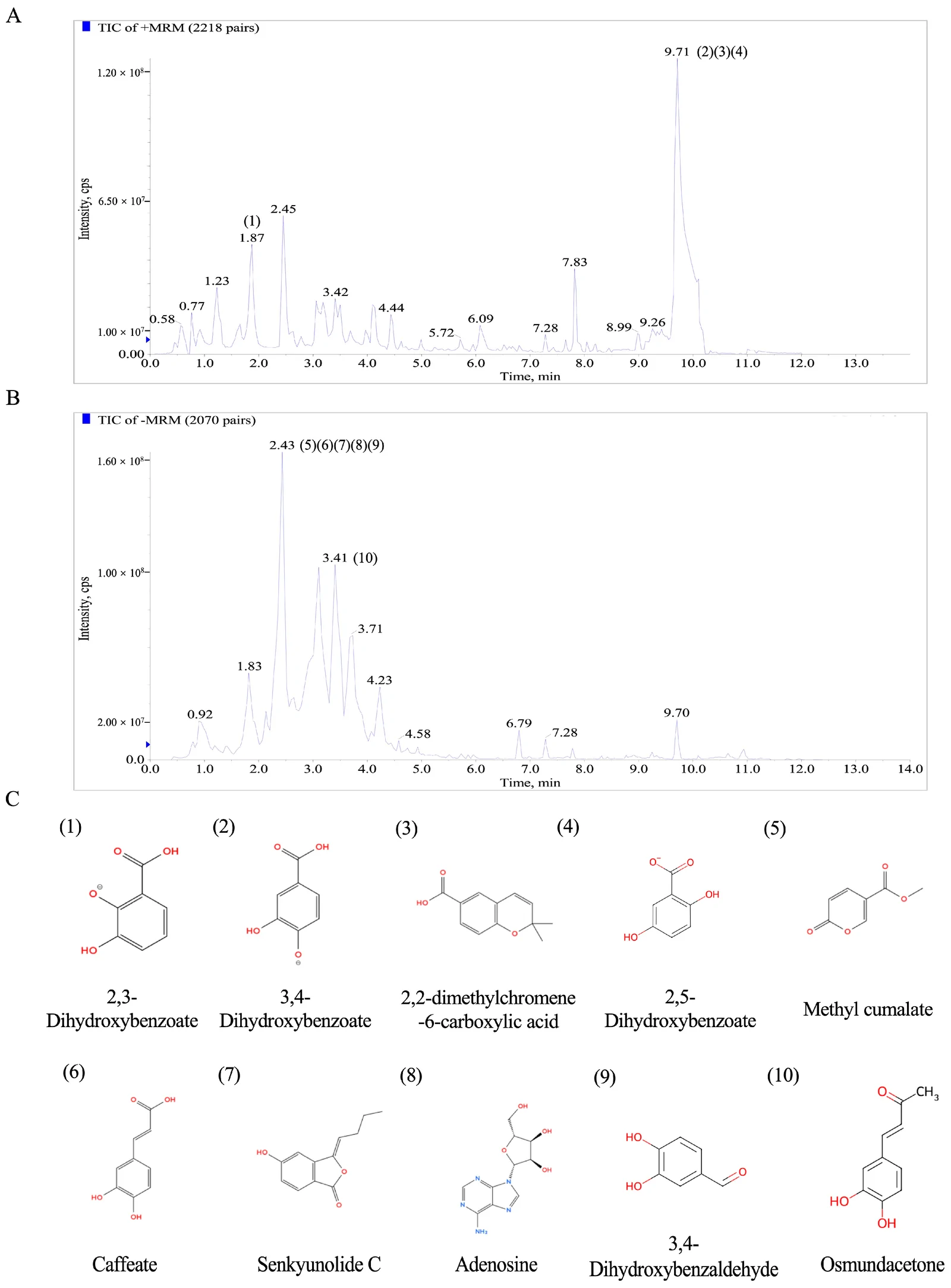
Content Determination and Component Identification in IO Extracts. (**A**,**B**) UPLC-ESI-MS/MS total ion chromatograms in positive and negative modes. The small blue triangle at the origin indicates the chromatogram start point (time zero). (**C**) Top 10 compounds identified at Level 1 confidence.

The results showed that the polysaccharide content was 19.67% ± 0.32; uronic acid content was 7.56% ± 0.18; reducing sugar content was 12.78% ± 0.85; total protein content was 7.55% ± 0.46; and total phenolic content was 12.98% ± 0.32. Polysaccharides were the most abundant component among these (Table 1). Furthermore, the top 10 components in IO extract identified by UPLC-ESI-MS/MS at Level 1 confidence are adenosine, diisobutyl phthalate, 2,2-dimethylchromene-6-carboxylic acid, senkyunolide C, 2,3-dihydroxybenzoate, 3,4-dihydroxybenzoate, gamma-resorcylate, 2,5-dihydroxybenzoate, methyl cumalate, and caffeate (Figure 1A–C).

### 3.2. IO Reduces Serum Bilirubin Levels and Alleviates Liver Injury in Mice with Hemolytic Jaundice

**Figure 2:**
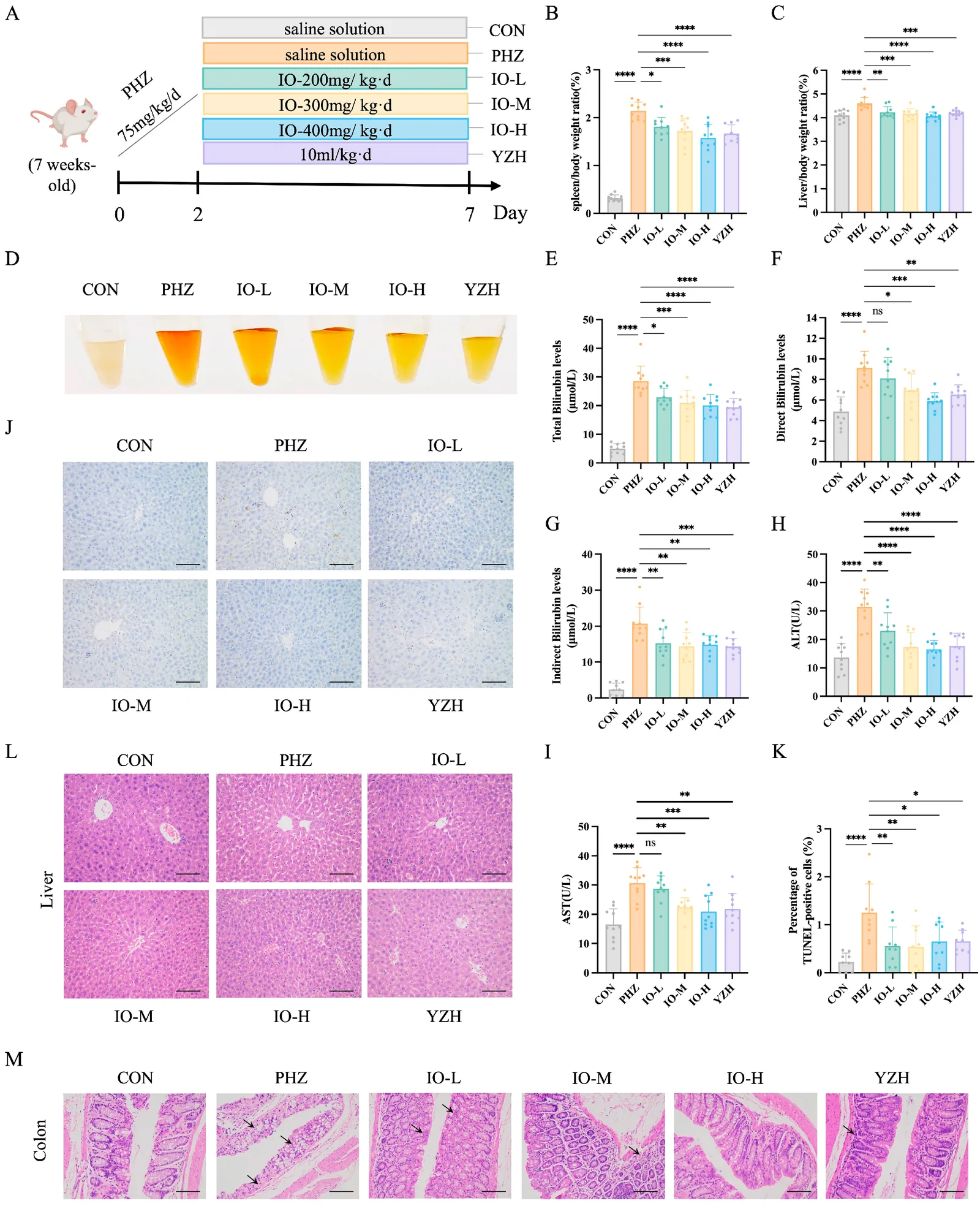
IO ameliorates hemolytic jaundice in mice by reducing serum bilirubin and providing hepatoprotection. (**A**) Schematic diagram of the animal experiment. (**B**,**C**) Spleen index and Liver index (*n* = 10). (**D**) Representative photographs of serum appearance. (**E**–**G**) Serum total bilirubin, direct bilirubin, and indirect bilirubin levels (*n* = 10). (**H**,**I**) Serum ALT and AST levels (*n* = 10). (**J**) TUNEL staining in the liver, scale bar = 100 μm. (**K**) Quantitative analysis of TUNEL-positive cells in the liver (*n* = 9). (**L**,**M**) H&E staining in the liver and intestine, scale bar = 100 μm. The black arrowheads indicate the sites of injury. * *p* < 0.05, ** *p* < 0.01, *** *p* < 0.001, **** *p* < 0.0001. ns = not significant.

The animal model was established following the schematic illustrated in Figure 2A. In contrast to the CON group, the PHZ group exhibited marked increases in spleen and liver indices, which were dose-dependently reversed by IO treatment (Figure 2B,C). Macroscopically, IO treatment alleviated the PHZ-induced serum darkening, which served as an initial indicator of hyperbilirubinemia (Figure 2D). Quantitative measurements of serum TBIL, DBIL, and IBIL further confirmed these observations, and collectively these findings suggest that IO reduces PHZ-induced bilirubin elevation (Figure 2E–G).

Hemolytic jaundice imposes a significant metabolic burden on the liver, often leading to secondary injury. To determine whether IO has hepatoprotective potential, we measured serum ALT and AST, established biomarkers of hepatic injury. PHZ-induced hemolytic jaundice was associated with marked ALT/AST elevations, which were dose-dependently alleviated by IO (Figure 2H,I). TUNEL and H&E staining further confirmed that IO alleviated hepatocyte apoptosis and histopathological damage (Figure 2J–L).

Indirect bilirubin generated from hemolysis is directly toxic to the intestinal mucosa, leading to gut microbiota dysbiosis and impairment of intestinal barrier function. To further evaluate whether IO could alleviate intestinal injury under these conditions, we performed H&E staining of colonic tissue sections. The results showed that PHZ-induced hemolytic jaundice caused marked colonic injury, as evidenced by multiple areas of epithelial detachment and focal glandular necrosis replaced by connective tissue in the lamina propria. In contrast, IO treatment significantly attenuated this intestinal damage, with only occasional epithelial detachment, preserved glandular architecture, and abundant goblet cells. Collectively, these findings demonstrate that IO not only reduces bilirubin elevation but also exerts both hepatoprotective and intestinal protective effects during hemolytic jaundice.

### 3.3. IO Alters the Gut Microbiota Composition in Mice with Hemolytic Jaundice

**Figure 3:**
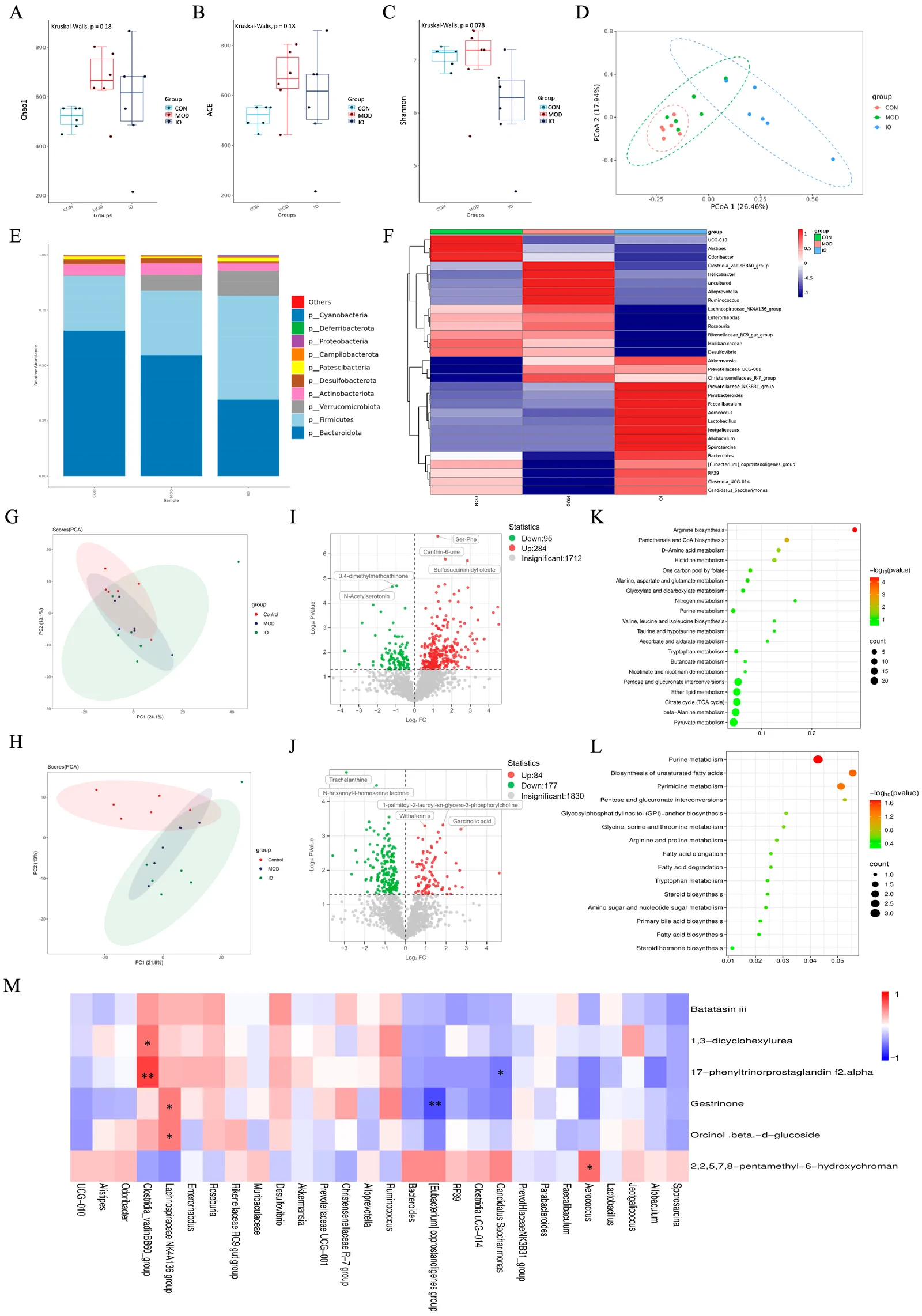
IO alters the gut microbiome and metabolites in hemolytic jaundice. (**A**–**C**) Alpha diversity indices, including Chao1 (**A**), ACE (**B**), and Simpson (**C**) indices, among different experimental groups. (**D**) PCoA based on Bray–Curtis dissimilarity. (**E**) Relative abundance of the dominant bacterial taxa at the phylum level across groups. (**F**) Heatmap showing the relative abundance of the dominant bacterial genera across groups. (**G**,**H**) PCA plot in positive and negative ion mode. (**I**,**J**) Volcano plot of differential metabolites for CON vs. PHZ (**C**) and IO vs. PHZ (**D**). (**K**,**L**) KEGG enrichment of differential metabolites for CON vs. PHZ (**E**) and IO vs. PHZ (**F**). (**M**) Correlation heatmap between differential bacterial genera and differential metabolites. * p < 0.05, ** p < 0.01.

Since polysaccharides constitute the major fraction of IO extract and are known to modulate gut microbiota, we performed 16S rRNA sequencing to determine whether IO affects gut microbiota. 16S rRNA sequencing revealed that PHZ-induced hemolytic jaundice was associated with increased Chao1, ACE, and Simpson indices, suggesting elevated microbial richness and diversity (Figure 3A–C). IO treatment partially attenuated these increases. Principal coordinates analysis (PCoA) showed distinct clustering among the CON, PHZ, and IO groups, indicating significant shifts in gut microbial composition (Figure 3D). At the phylum level, *Bacteroidota*, *Firmicutes*, *Verrucomicrobiota*, and *Actinobacteriota* were the dominant taxa. The relative abundance of *Bacteroidota* decreased in the PHZ group and further declined following IO treatment, whereas *Firmicutes* abundance was notably higher in the IO group than in both the CON and PHZ groups (Figure 3E). At the genus level, IO treatment modulated several taxa altered by model induction, including *Ruminococcus* and *RF39*, and increased the abundance of potentially beneficial genera, such as *Lactobacillus* (Figure 3F).

### 3.4. Effects of IO on Fecal Metabolites in Mice with Hemolytic Jaundice

The function of the gut microbiota is closely tied to the activity of its metabolites. We conducted a metabolomic analysis on fecal samples from the CON, PHZ, and IO groups. Principal component analysis (PCA) plots under both positive and negative ion modes demonstrated distinct clustering and significant differences in metabolite profiles among the groups (Figure 3G,H). PHZ treatment significantly altered 95 downregulated and 284 upregulated metabolites versus CON (Figure 3I), whereas IO altered 84 upregulated and 177 downregulated metabolites versus PHZ (Figure 3J). Volcano plots identified candidate biomarkers such as 3,4-dimethylmethcathinone and sulfosuccinimidyl oleate for PHZ, and trachelanthine and *N*-hexanoyl-L-homoserine lactone for IO (Figure 3I,J). KEGG analysis showed that PHZ-affected metabolites were enriched in arginine, pantothenate, and CoA biosynthesis (Figure 3K), whereas IO-affected metabolites were linked to purine and unsaturated fatty acid metabolism (Figure 3L). Correlation analysis between differential metabolites and the top 20 altered genera revealed notable associations (Figure 3M), including positive correlations of *Clostridia_vadinBB60*_group with 1,3-dicyclohexylurea and 17-phenyltrinorprostaglandin F2.alpha, and of *Lachnospiraceae_NK4A136*_group with gestrinone and orcinol β-D-glucoside.

### 3.5. Antibiotic Intervention Abrogates the Protective Effects of IO in Hemolytic Jaundice

**Figure 4:**
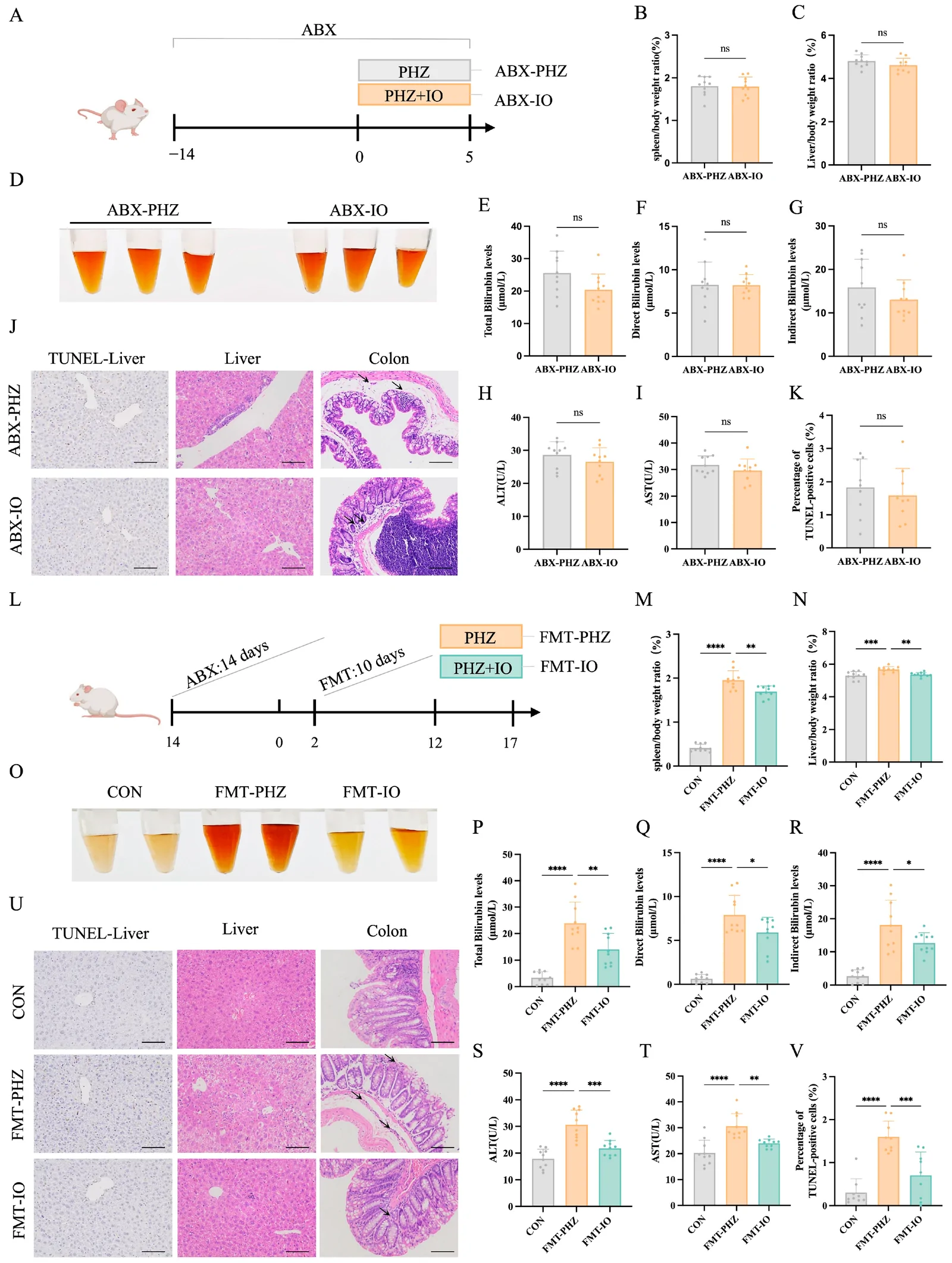
IO attenuates hemolytic jaundice via gut microbiota-dependent mechanisms as revealed by antibiotic intervention and FMT. (**A**) Schematic diagram of antibiotic intervention animal experimental design. (**B**,**C**) Spleen index and liver index (*n* = 10). (**D**) Representative photographs of serum appearance. (**E**–**G**) Serum total bilirubin, direct bilirubin, and indirect bilirubin levels (*n* = 10). (**H**,**I**) Serum ALT and AST levels (*n* = 10). (**J**) TUNEL staining (left panel) and H&E staining (right panel) of liver and intestinal tissues, scale bar = 100 μm. The black arrowheads indicate the sites of injury. (**K**) Quantitative analysis of TUNEL-positive cells in the liver (*n* = 9). (**L**) Schematic diagram of FMT animal experimental design. (**M**,**N**) Spleen index and liver index (*n* = 10). (**O**) Representative photographs of serum appearance. (**P**–**R**) Serum total bilirubin, direct bilirubin, and indirect bilirubin levels (*n* = 10). (**S**,**T**) Serum ALT and AST levels (*n* = 10). (**U**) TUNEL staining (left panel) and H&E staining (right panel) of liver and intestinal tissues, scale bar = 100 μm. The black arrowheads indicate the sites of injury. (**V**) Quantitative analysis of TUNEL-positive cells in the liver (*n* = 9). * p < 0.05, ** p < 0.01, *** p < 0.001, **** p < 0.0001. ns = not significant.

In previous experiments, we confirmed significant alterations in the gut microbiota following IO treatment and their correlation with metabolite changes. To further validate the role of gut microbiota, we performed a gut microbiota depletion experiment in mice (Figure 4A). The liver index and spleen index exhibited no significant differences between the ABX-PHZ and ABX-IO groups (Figure 4B,C). Macroscopic examination of serum appearance revealed similar color between the two groups (Figure 4D). Subsequent measurements of serum TBIL, DBIL, and IBIL levels showed no significant differences (Figure 4E–G). Biochemical analysis indicated that the ability of IO to reduce serum ALT and AST levels was abolished after antibiotic treatment (Figure 4H,I). This finding was further corroborated by the results from TUNEL staining (Figure 4J,K) and H&E staining (Figure 4J) of liver tissues. Additionally, H&E staining of the colon (Figure 4J) showed that the ABX-PHZ group exhibited epithelial detachment and glandular necrosis, while the ABX-IO group displayed similar colonic damage with no apparent improvement. These findings indicated that the protective effect of IO against intestinal tissue damage was eliminated following antibiotic administration.

### 3.6. Fecal Microbiota Transplantation from IO-Treated Mice Elicits Effects Similar to Direct IO Administration

To validate the findings, we performed an additional fecal microbiota transplantation (FMT) experiment in mice (Figure 4L). Compared to the CON group, the FMT-PHZ group showed increased liver and spleen indices, whereas the FMT-IO group exhibited a significant reduction in these indices compared to the FMT-PHZ group (Figure 4M,N). Macroscopic observation of serum appearance revealed darker coloration in the FMT-PHZ group, which was notably lighter in the FMT-IO group (Figure 4O). Further measurement of serum TBIL, DBIL, and IBIL levels showed significant elevations in the FMT-PHZ group, while the FMT-IO group demonstrated a marked decrease compared to FMT-PHZ (Figure 4P–R). Biochemical analysis indicated that the FMT-IO group similarly resulted in reduced serum ALT and AST levels (Figure 4S,T). In line with these findings, liver TUNEL staining (Figure 4U,V) and H&E staining (Figure 4U) corroborated the observed improvements. Furthermore, H&E staining of colon tissue (Figure 4U) indicated that the FMT-IO group also conferred a protective effect against intestinal tissue damage, as evidenced by only minimal epithelial detachment, preserved glandular architecture, and increased goblet cell numbers compared to the PHZ group.

### 3.7. Effect of IO on the Gut Metagenomic Profile in Mice with Hemolytic Jaundice

**Figure 5:**
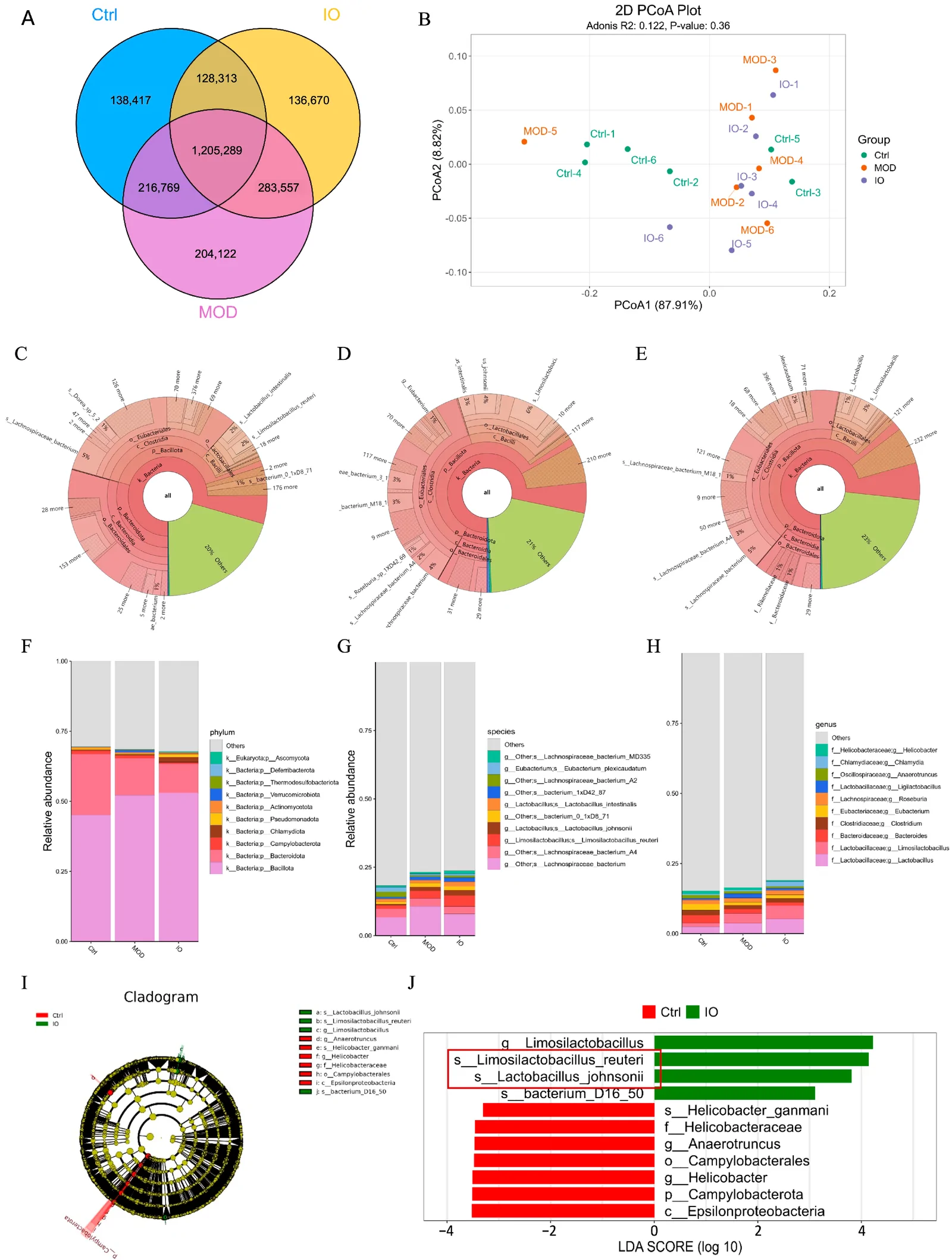
Impact of IO on the gut metagenomic profile in mice with hemolytic jaundice. (**A**) Venn diagram of the number of genes across groups. (**B**) PCoA based on Bray–Curtis dissimilarity. (**C**–**E**) Relative abundance of the dominant bacterial taxa at the phylum (**C**), genus (**D**), and species (**E**) levels. (**F**–**H**) Species composition analysis of the CON (**F**), PHZ (**G**), and IO (**H**) group. (**I**) LEfSe analysis. (**J**) LDA scores (LDA score > 3).

To identify the specific core microbiota responsible for the therapeutic effects of IO, fecal samples from the CON, PHZ, and IO groups were subjected to metagenomic sequencing. This approach allows for the resolution of microbial alterations at the species or even strain level. A total of 1,688,788 genes were identified in the CON group, 1,909,737 in the PHZ group, and 1,753,829 in the IO group (Figure 5A). PCoA revealed significant differences in the composition and distribution of gut microbial species among the groups (Figure 5B). Figure 5C–E illustrate the species composition of the CON, PHZ, and IO groups, respectively. Figure 5F–H illustrate alterations in gut microbiota at the phylum, genus, and species levels across all groups. The relative-abundance bar plot demonstrated that the proportion of *Bacillota* was elevated in the MOD group compared with the CON group, and further increased in the IO group, whereas the relative abundance of Bacteroidetes was markedly decreased in the IO group (Figure 5F). These observations indicated that IO extract did not simply restore gut microbiota balance at the phylum level; instead, it drove a distinct microbial shift. At the genus level, IO extract partially reversed PHZ-induced alterations of several genera including *Clostridium* and *Eubacterium* (Figure 5G). Meanwhile, the abundance of potential beneficial genera such as the probiotic *Lactobacillus* was increased following IO intervention (Figure 5H). Collectively, these data confirmed substantial compositional shifts in gut microbiota among the CON, MOD and IO groups. Evolutionary branching diagrams of differential species (Figure 5I) and histogram of Linear Discriminant Analysis (LDA) scores (Figure 5J) aided in screening species that were significantly different between groups. Compared to the PHZ group, the primary species altered in the IO group included *Limosilactobacillus reuteri* and *Lactobacillus johnsonii* which are highlighted by the red box in Figure 5J.

### 3.8. Effects of FMT-IO on SCFAs in Mice with Hemolytic Jaundice

**Figure 6:**
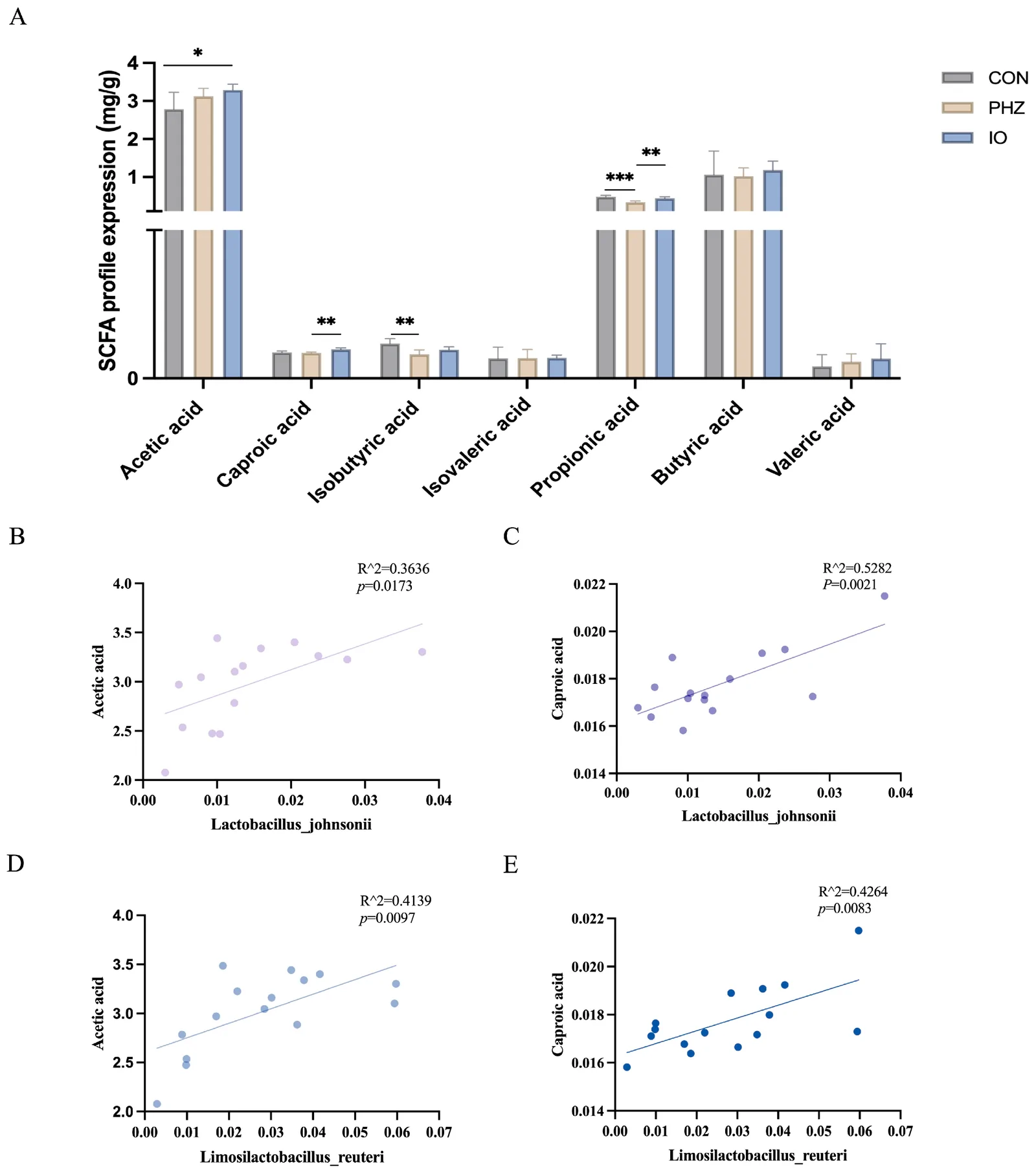
Effects of FMT-IO on SCFAs in Mice with Hemolytic Jaundice. (**A**) Fecal SCFA levels. (**B**,**C**) Correlation analysis between *Lactobacillus johnsonii* and acetic/caproic acid. (**D**,**E**) Correlation analysis between *Limosilactobacillus reuteri* and acetic/caproic acid. * p < 0.05, ** p < 0.01, *** p < 0.001.

SCFAs are closely associated with the gut microbiota [37]. To investigate whether hemolytic jaundice induces alterations in SCFAs and the potential regulatory role of IO, we measured the levels of SCFAs in the colonic contents of mice from the CON, PHZ, and IO groups. Compared with the CON group, the PHZ group showed significantly decreased levels of propionic acid and isobutyric acid, while the IO group exhibited a significant increase in acetic acid. In contrast to the PHZ group, the IO group demonstrated significantly elevated levels of propionic acid and caproic acid (Figure 6A). Correlation analysis between SCFAs alterations and microbial composition changes revealed that *Limosilactobacillus reuteri* and *Lactobacillus johnsonii* were significantly correlated with the variations in acetic acid and caproic acid (Figure 6B–E).

### 3.9. IO Influences the Enterohepatic Circulation Through Upregulation of the FXR/PXR-BSEP/MRP2 Pathway

**Figure 7:**
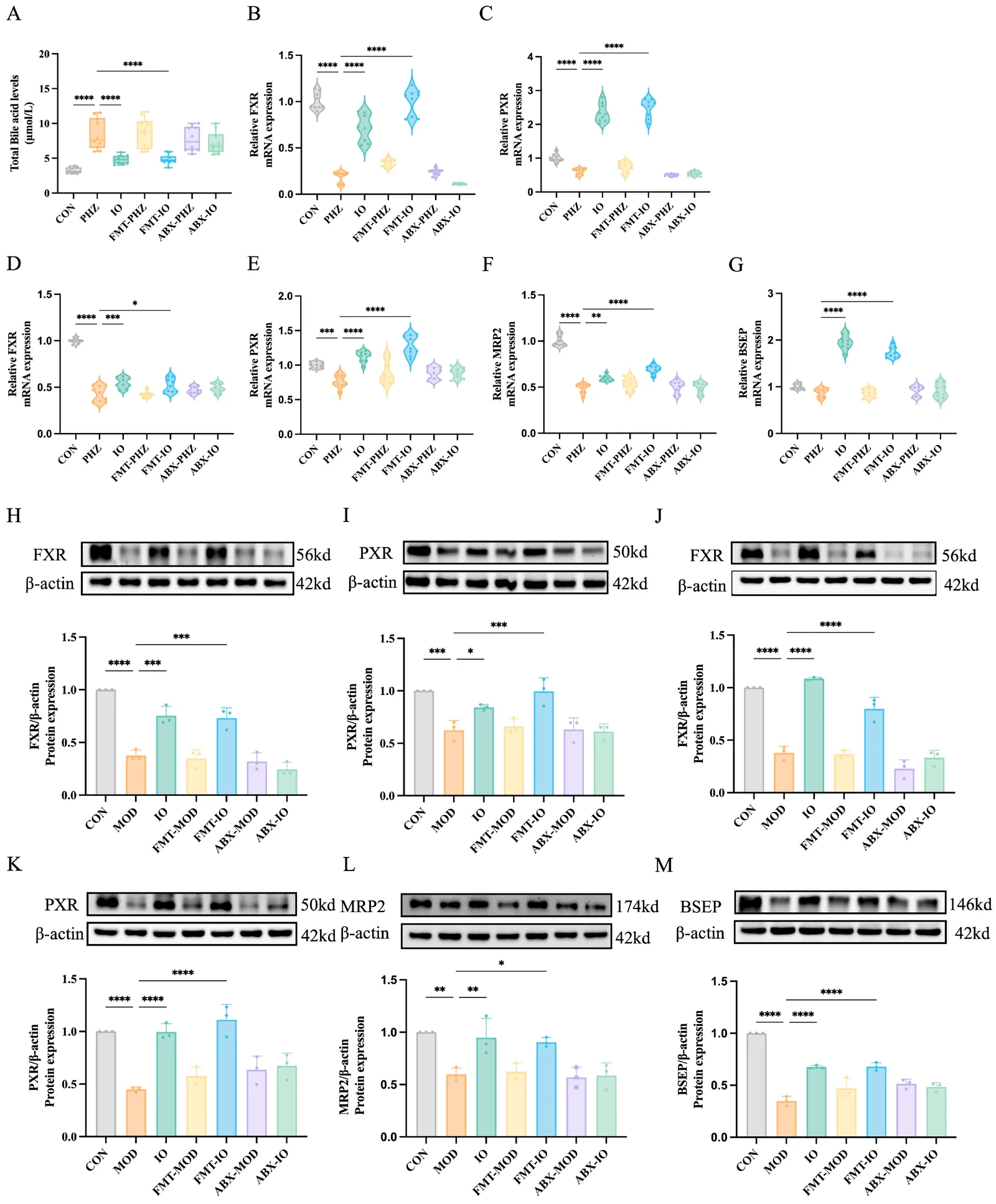
IO reduces serum bilirubin and alleviates liver injury by promoting bile acid metabolism. (**A**) Serum bile acid concentration across groups. (**B**,**C**) Relative mRNA expression levels of PXR and FXR in the colon. (**D**–**G**) Relative mRNA expression levels of Mrp2, PXR, FXR, and BSEP in the liver. (**H**,**I**) Relative protein expression levels of PXR and FXR in the colon. (**J**–**M**) Relative protein expression levels of FXR, PXR, Mrp2, and BSEP in the liver. * p < 0.05, ** p < 0.01, *** p < 0.001, **** p < 0.0001.

Bile acid metabolism is closely associated with the gut microbiota. Bile acids exert influence on gut microbiota composition, which in a reciprocal manner regulates the bile acid pool’s size and composition, thereby modulating metabolism systemically [38]. We first measured the total bile acid content in serum. The level was elevated in the PHZ group, indicating impaired biliary excretion, while it was reduced in the IO and FMT-IO groups, suggesting that IO promoted the excretion of bile acid metabolites through modulation of the gut microbiota (Figure 7A).

FXR and PXR are important nuclear receptors and sensors in the liver and intestine. FXR is the primary regulator of bile acid homeostasis and can directly upregulate the expression of hepatic BSEP to promote bile acid excretion [39]. PXR is the primary sensor for xenobiotics; its activation primarily upregulates MRP2 and exerts a complex and indirect influence on BSEP [40,41].

To evaluate the impact of IO on bile acid metabolism, we examined the gene and protein expression of FXR and PXR in both the liver (Figure 7D,E,J,K) and intestine (Figure 7B,C,H,I), as well as the hepatic transporters BSEP and MRP2 (Figure 7F,G,L,M). In the PHZ, FMT-MOD, ABX-MOD, and ABX-IO groups, the expression of FXR, PXR, BSEP, and MRP2 was consistently downregulated at both the transcriptional and translational levels. In contrast, the IO and FMT-IO groups exhibited upregulated expression of these genes and proteins. Collectively, these findings suggest that IO modulates the enterohepatic circulation by upregulating FXR and PXR expression in the liver and intestine, which in turn enhances hepatic BSEP and MRP2 expression, facilitating the elimination of bile acids and bilirubin and contributing to the alleviation of liver injury.

## 4. Discussion

Jaundice is a common condition among neonates, primarily attributed to increased bilirubin production, limited processing capacity of hepatocytes, and enhanced enterohepatic circulation of bilirubin [10]. Hemolytic jaundice, which arises from hemolytic disease of the newborn, is the leading cause of pathological jaundice in this demographic [42]. Given the lack of well-established pharmacological treatments in clinical practice, traditional interventions such as phototherapy and exchange transfusion are associated with certain limitations and potential adverse effects Therefore, the exploration of effective pharmacological strategies for hemolytic jaundice as adjunctive and complementary therapies is critically important. Probiotic supplementation has shown potential in managing this condition [43]. *Inonotus obliquus*, a medicinal fungus, has exhibited hepatoprotective effects and regulatory actions on gut microbiota in preliminary studies [44,45]. However, the therapeutic role and underlying mechanisms of IO in the context of hemolytic jaundice require further elucidation. To address this research gap, the present study utilized a well-established model of hemolytic jaundice, induced by PHZ, to explore the underlying mechanisms by which IO ameliorates hemolytic jaundice. The findings demonstrate that IO significantly mitigates the hemolysis-induced elevation in serum bilirubin levels and confers hepatoprotective effects. This regulatory mechanism is predominantly linked to IO’s modulation of bile acid metabolism and its modulation of the bilirubin “gut microbiota-metabolite-enterohepatic circulation” axis.

The relationship between bilirubin metabolism and bile acid metabolism is complex. Obstruction of bile flow impedes the proper excretion of conjugated bilirubin into the bile ducts, resulting in its reflux into the bloodstream and exacerbating hyperbilirubinemia [2]. Additionally, dysregulation of bile acid metabolism leads to the accumulation of hydrophobic bile acids, such as CDCA and DCA, which are cytotoxic due to their induction of mitochondrial dysfunction, oxidative stress, and apoptosis in hepatocytes [46]. We hypothesized that this cell damage might affect the expression of enzymes related to bilirubin and bile acid metabolism. Therefore, we quantified bile acid levels in the blood and observed a significant increase in serum bile acid levels post-modeling, indicative of reflux into the bloodstream due to impaired bile acid excretion. Treatment with IO significantly decreased serum bile acid levels. Furthermore, we assessed the hepatic bile acid transporters BSEP and MRP2, noting a decrease in their expression post-modeling and an increase following IO treatment. This suggests that IO can stimulate the expression of bile acid excretory proteins, thereby facilitating the transport of bile acids from the liver to the intestines.

PXR and FXR are critical nuclear receptors predominantly expressed in the intestine and liver, playing a significant role in bile acid metabolism [47]. FXR, as the principal bile acid receptor, orchestrates the absorption and reabsorption of bile acids within the intestine, thereby ensuring bile acid homeostasis [48]. Conversely, PXR acts as a xenobiotic sensor, facilitating the clearance of toxic substances and contributing to bile acid metabolism [49]. Prior research indicated that the activation of PXR and FXR mediates the induction of BSEP and MRP2 expression [50]. In our investigation, we observed increased expression levels of both PXR and FXR in the liver and intestine subsequent to IO administration. This finding suggests that IO may have an impact on bile acid metabolism, a process intricately linked to enterohepatic circulation. The modulation of receptor expression may be influenced by the gut microbiota; however, further research is necessary to elucidate the underlying mechanisms.

Our experimental results indicate that depleting the gut microbiota abolishes the receptor-activating effects of IO, whereas transplanting feces from the IO-treated group into mice produces effects similar to direct IO administration. This verifies the crucial role of the gut microbiota. Additionally, our findings suggest that a decrease in intestinal bile acid flow induced by PHZ alters the intestinal microenvironment, including the gut microbial composition and intestinal motility. This shift may enhance the dissociation of bilirubin in the intestine, promoting its reabsorption into the liver and thereby increasing the bilirubin burden on the already compromised liver. Hence, the modulation of bile acid metabolism is one of the key mechanisms through which IO exerts its bilirubin-lowering effects.

Based on integrated 16S rRNA sequencing and metagenomic analysis, we found that the gut microbiota composition underwent significant changes after IO treatment. At the phylum level, *Bacteroidetes* abundance was lower in the PHZ group than in the CON group and was further reduced in the IO group. In contrast, the proportion of *Firmicutes* in the IO group increased significantly compared to both the CON and PHZ groups. This indicates that, at the phylum level, the regulatory effect of the IO extract does not represent a restoration of balance but rather a more intriguing “extreme” shift. At the genus level, the IO significantly enhanced the abundance of several potentially beneficial genera, notably the well-established probiotic genus *Lactobacillus*. Specifically, the relative abundances of *Lactobacillus johnsonii* and *Limosilactobacillus reuteri* were greatly increased, and their enrichment was correlated with the alleviated phenotype of hemolytic jaundice. It is critical to emphasize that such omics-based association results cannot establish causal relationships between the two strains and the hepatoprotective effects of IO. Differential abundance merely reflects statistical correlation, which may be interfered by other co-variant microorganisms or secondary metabolic changes induced by improved liver function. For this reason, we only define these two *lactobacilli* as candidate functional strains rather than confirmed direct mediators of bilirubin clearance. Further gnotobiotic mouse mono-colonization and single-probiotic supplementation experiments are required to distinguish correlation from causation, which will be carried out in our follow-up research.

Accumulated published data have illustrated the characteristic biological functions of *L. reuteri* and *L. johnsonii* in regulating bile acid metabolism and alleviating liver injury. *Lactobacillus johnsonii* possesses high bile salt hydrolase activity [51], which catalyzes the deconjugation of conjugated bile acids, remodels intestinal bile acid pools and further activates hepatic FXR signaling to relieve cholestatic liver damage [52]. As a classic commensal probiotic, *Limosilactobacillus reuteri* produces reuterin to suppress pathogenic bacteria, repairs intestinal mucosal barrier, and reduces hepatic inflammatory infiltration via inhibiting TLR4/NF-κB pathway [53]. Beyond modulating bile acid composition, metabolites secreted by these *lactobacilli* are also capable of shaping intestinal microenvironment and regulating enterohepatic signaling crosstalk [45]. Additionally, strain-specific functional discrepancies should not be ignored, as biological effects often vary across different isolates of the same species [54]. In addition, recent hepatic studies confirm that elevated abundance of *Limosilactobacillus reuteri* can boost intestinal free bile acid content, upregulate hepatic bile acid transporters through FXR pathway, and accelerate systemic bilirubin excretion [55]. The above evidence provides reasonable mechanistic clues for the potential participation of the two strains in IO-mediated remission of hemolytic jaundice, while definitive causal evidence remains absent in the current study.

We further investigated how gut microbiota influences receptors via metabolites. SCFAs are the end products of dietary fiber metabolism by gut microbiota [56]. After administration of IO, we observed significant increases in acetic acid, propionic acid, and caproic acid. Correlation analysis indicated a positive relationship between changes in the abundance of *Lactobacillus johnsonii* and *Limosilactobacillus reuteri* with the levels of acetic acid and caproic acid. Acetic acid accounts for 50–60% of SCFAs and serves as an important substrate for the synthesis of cholesterol and fatty acids in the human body [57]. Propionic acid can modulate systemic metabolism, participates in hepatic gluconeogenesis, regulates lipid metabolism, and reduces the risk of cardiovascular diseases [58]. Although caproic acid is present in small amounts, studies have shown that it can cross the blood–brain barrier and affect the brain, acting as a key messenger in the gut-brain axis [59]. IO may exert beneficial effects on the body by enriching specific SCFAs and improve hemolytic jaundice through the “gut microbiota–short-chain fatty acid” axis.

Most existing investigations of IO extracts mainly focus on anti-diabetic, hypolipidemic, and anti-tumor models. For gut microbial detection, these studies only rely on 16S rRNA gene sequencing to describe rough flora variation at the phylum level, without metagenomic and metabolomic validation of functional metabolites such as bile acids and SCFAs [45,60]. A large proportion of existing studies merely describe alterations in microbial composition, yet lack functional verification to unravel the causal relationship between flora shifts and host phenotypic changes [33]. Moreover, most relevant experiments adopt crude extracts of *Inonotus obliquus*, making it difficult to identify which active constituent contributes to gut microbiota remodeling [61]. Additionally, few works further explore how gut microbiota mediates the hepatoprotective bioactivities of IO at the enterohepatic axis level. Very few reports construct hemolytic jaundice or hyperbilirubinemia animal models, and nearly no prior work integrates antibiotic-mediated gut microbiota depletion and FMT to functionally verify that intact gut microbiota is indispensable for IO’s protective effects.

Compared with previous literature, our work innovatively combines multi-omics analysis, microbial ablation, and FMT assays to systematically construct the “IO–gut microbiota–bile acid–FXR/PXR–bilirubin excretion” regulatory axis specific to hemolytic liver injury. Nevertheless, consistent with most research on natural crude fungal extracts, this study still has unavoidable limitations: we did not separate and purify single bioactive compounds of IO to clarify which ingredient drives gut microbiota remodeling; meanwhile, the causal relationship between candidate lactobacilli and jaundice relief remains unvalidated. These gaps provide clear directions for our subsequent deeper mechanistic research.

**Figure 8:**
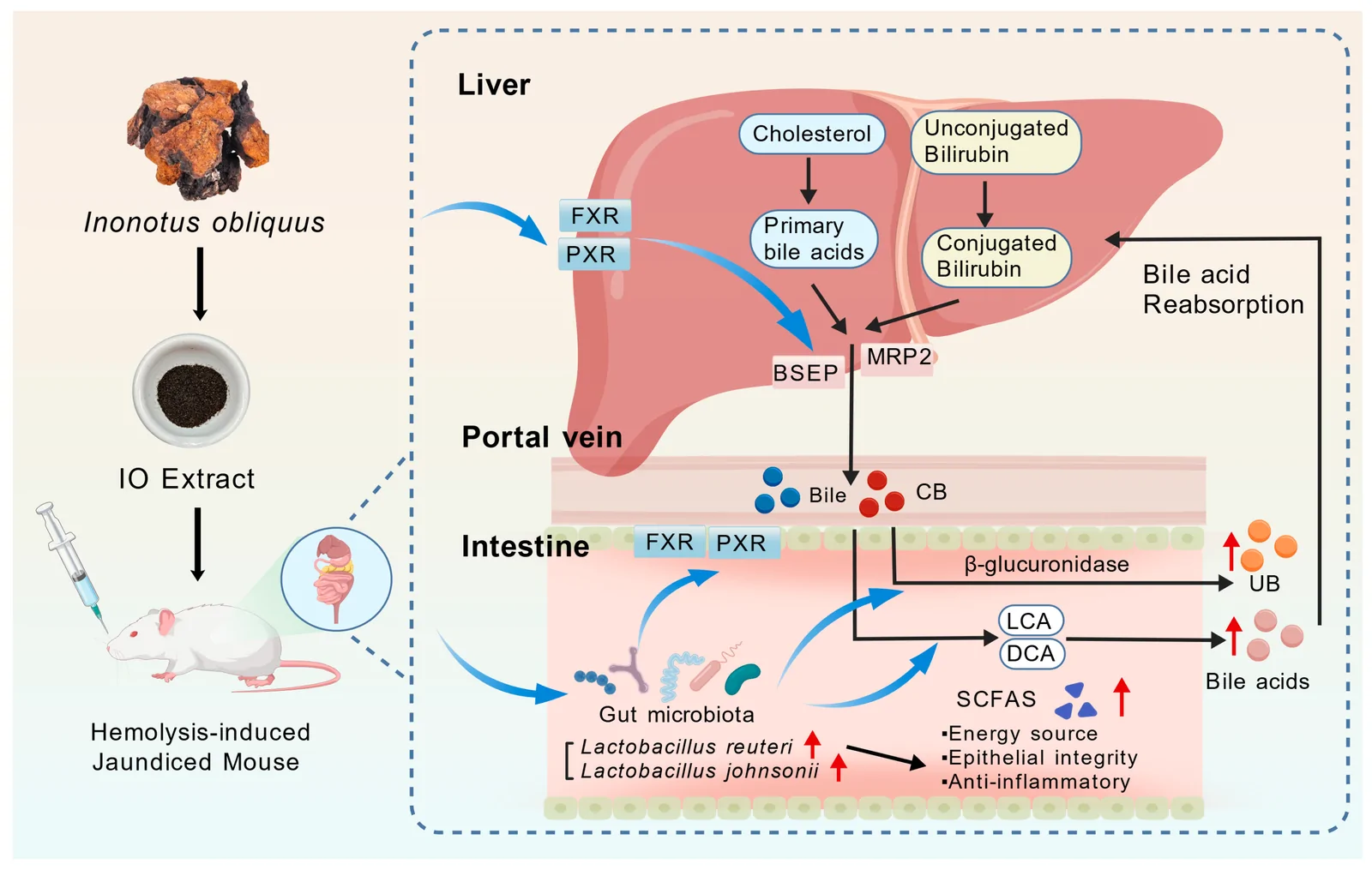
Possible mechanisms for IO reduce bilirubin and alleviate liver injury. Red arrowheads indicate upregulation; blue arrowheads indicate the direction of regulation.

While the pathophysiological context of neonatal hemolytic jaundice markedly differs from adult hemolysis, the applicability of findings derived from adult models to neonatal hemolytic jaundice is limited, especially concerning hepatic bilirubin metabolism, albumin binding capacity, and the maturation of the blood–brain barrier. Consequently, the therapeutic implications of these findings for neonatal hyperbilirubinemia necessitate further validation in appropriate neonatal models. The formal experiment was confined to two groups, ABX-PHZ and ABX-IO. While this design choice facilitated the simplification of experimental conditions, it concurrently constrained the ability to make direct comparisons with other potential variables, thus representing a limitation of the study. The incomplete factorial design of the antibiotic intervention prevents the drawing of definitive conclusions regarding the independent effects of each antibiotic. The engraftment of FMT was not directly verified, and the observed correlations between FXR/PXR activity and transporter expression do not establish causality. Furthermore, the causal roles of specific microbial strains were not investigated. Nevertheless, the robustness and consistency of the results across various experiments strongly support the central conclusion of this research. In our study, we detailed the effects of the IO on hemolytic jaundice. Although polysaccharides are a major component, leading us to focus our investigation on the gut microbiota—where we validated its role and identified a potential mechanism involving bile acid metabolism—the complexity of the extract’s composition suggests it may exert multi-target and multi-mechanism effects. In this study, we did not separate, purify, or individually assess the impact of specific components within the IO. Additionally, while metagenomics identified significantly enriched beneficial gut bacteria, *Lactobacillus johnsonii* and *Limosilactobacillus reuteri,* and proposed them as potential core mediators of IO’s efficacy, we did not conduct in vitro isolation and culture experiments for further validation. These aspects will be the focus of our subsequent research (Figure 8).

## 5. Conclusions

In conclusion, this study successfully isolated an extract from IO utilizing the water extraction and alcohol precipitation technique. The primary constituents of the extract were characterized using physicochemical analyses and UPLC-MS/MS. The experimental findings indicate that the extract possesses hepatoprotective properties and bilirubin-lowering effects in the context of hemolytic jaundice. Notably, the high-dose group (400 mg/kg/day) exhibited the most significant therapeutic efficacy. Mechanistic investigations that integrate gut microbiota analysis with metabolomic profiling have elucidated two principal pathways. Firstly, IO activates both hepatic and intestinal PXR and FXR receptors, resulting in the upregulation of the efflux transporters BSEP and MRP2. This activation facilitates the excretion of bile acids and bilirubin from the liver into the intestine, thereby mitigating the risk of hepatotoxicity associated with their intrahepatic accumulation. Secondly, IO induces beneficial alterations in the gut microbiota composition by increasing the prevalence of putative probiotic species, such as *Lactobacillus johnsonii* and *Limosilactobacillus reuteri*. These microbial alterations lead to increased levels of short-chain fatty acids and modified metabolite profiles, which subsequently enhance the intestinal breakdown of bilirubin and bile acids while inhibiting their reabsorption. Collectively, these coordinated processes contribute to the reduction in serum bilirubin levels and the enhancement of hepatoprotective functions, indicating that IO holds potential for application in the treatment of jaundice and the promotion of liver health.

## Figures and Tables

**Table 1:** Results of determination of the main components of IO.

Components	Component Content (%)	Quantification Method
Polysaccharide	19.67 ± 0.32	Phenol-Sulfuric Acid Method
Uronic acid	7.56 ± 0.18	Carbazole-Sulfuric Acid Method
Reducing sugar	12.78 ± 0.85	3,5-Dinitrosalicylic Acid Method
Total protein	7.55 ± 0.46	BCA Method
Total phenol	12.98 ± 0.28	Folin–Ciocalteu Method

Data represent mean ± standard deviation (*n* = 3). All contents are calculated based on the weight of dried *Inonotus obliquus* extract powder.

## Data Availability

The data that support the findings of this study are available in the Genome Sequence Archive (Genomics, Proteomics & Bioinformatics 2025) in National Genomics Data Center (Nucleic Acids Res 2025), China National Center for Bioinformation/Beijing Institute of Genomics, Chinese Academy of Sciences (GSA: CRA037136) that are publicly accessible at https://ngdc.cncb.ac.cn/gsa/browse/CRA037136 (accessed on 22 January 2026). Additional data that support the findings of this study are available from the corresponding author upon reasonable request.

## Supplementary Materials

The following supporting information can be downloaded at: https://www.mdpi.com/article/10.3390/nu18193225/s1, Table S1. Primer sequences for quantitative real-time PCR amplification.

## Abbreviations

The following abbreviations are used in this manuscript:

ABX	Antibiotic cocktail
ALT	Alanine aminotransferase
AST	Aspartate aminotransferase
BSEP	Bile salt export pump
CB	Conjugated bilirubin
CDCA	Chenodeoxycholic acid
DCA	Deoxycholic acid
DBIL	Direct bilirubin
FMT	Fecal microbiota transplantation
FXR	Farnesoid X receptor
GSA	Genome Sequence Archive
HDN	Hemolytic disease of the newborn
H&E	Hematoxylin and eosin
IBIL	Indirect bilirubin
IO	*Inonotus obliquus*
KEGG	Kyoto Encyclopedia of Genes and Genomes
LDA	Linear Discriminant Analysis
MRP2	Multidrug resistance-associated protein 2
NGDC	National Genomics Data Center
Ntcp	Na^+^-taurocholate cotransporting polypeptide
PCA	Principal component analysis
PCoA	Principal coordinates analysis
PHZ	Phenylhydrazine
PXR	Pregnane X receptor
SCFAs	Short-chain fatty acids
SD	Standard deviation
TBIL	Total bilirubin
TUNEL	Terminal deoxynucleotidyl transferase dUTP nick end labeling
UCB	Unconjugated bilirubin
UGT1A1	UDP-glucuronosyltransferase 1A1
UPLC-ESI-MS/MS	Ultra-performance liquid chromatography–electrospray ionization–tandem mass spectrometry
YZH	Yinzhihuang Oral Liquid
β-GD	β-glucuronidase
