# Supplementary material for: Multi-Omics Analysis Reveals the Potential Role of Inonotus obliquus in Mit-Igating Hemolytic Jaundice
Source: Nutrients. 2026 Sep 29;18(19):3225. doi: 10.3390/nu18193225 (PMC13667189; doi:10.3390/nu18193225)
Supplement: Supplementary file 1 [file nutrients-18-03225-s001.zip › nutrients-4458872-supplementary.pdf]

Supplementary Material

Article

Multi-Omics Analysis Reveals the Potential Role of *Inonotus obliquus* in Mit-Igating Hemolytic Jaundice

Fan Yang [1,†], Siyi Xie [1,†], Wenjing Yang [1,†], Chuanhong Zhu [1], Yurong Chen [1], Haozhuo Yang [1], Hongxia Yuan [2,*] and Qingshan Li [1,2,*]

Table S1

Primer sequences for quantitative real-time PCR amplification

Gene	Forward primers (5' - 3')	Reverse primers (5' - 3')
FXR	GGCAGAATCTGGATTTGGAATCG	GCTGAACTTGAGGAAACGGG
PXR	GATGGAGGTCTTCAAATCTGCC	GGCCCTTCTGAAAAACCCCT
BSEP	TCTGACTCAGTGATTCTTCGCA	CCCATAAACATCAGCCAGTTGT
MRP2	GCACTGTAGGCTCTGGGAAG	CATTTCCAAGTCTGGGAGGA
GAPDH	GGTTGTCTCCTGCGACTTCA	TGGTCCAGGGTTTCTTACTCC
